# Promotive effect of phytosulfokine - peptide growth factor - on protoplast cultures development in *Fagopyrum tataricum* (L.) Gaertn

**DOI:** 10.1186/s12870-023-04402-9

**Published:** 2023-08-10

**Authors:** Magdalena Zaranek, Reneé Pérez-Pérez, Anna Milewska-Hendel, Alexander Betekhtin, Ewa Grzebelus

**Affiliations:** 1 Institute of Biology, Biotechnology and Environmental Protection, Faculty of Natural Sciences, University of Silesia in Katowice, 28 Jagiellonska st, Katowice, 40-032 Poland; 2 Department of Plant Biology and Biotechnology, Faculty of Biotechnology and Horticulture, University of Agriculture in Krakow, al. Mickiewicza 21, Krakow, 31-120 Poland

**Keywords:** 2-aminoondane-2-phosphonic acid (AIP), Agarose, Driselase, Hypocotyl, Morphogenic callus, Non-morphogenic callus, Phenolic compounds, Plating efficiency, Polyvinylpyrrolidone (PVP), Tartary buckwheat

## Abstract

**Background:**

*Fagopyrum tataricum* (Tartary buckwheat) is a valuable crop of great nutritional importance due to its high level of bioactive compounds. Excellent opportunities to obtain plants with the high level or the desired profile of valuable metabolites may be provided by in vitro cultures. Among known in vitro techniques, protoplast technology is an exciting tool for genetic manipulation to improve crop traits. In that context, protoplast fusion may be applied to generate hybrid cells between different species of *Fagopyrum*. To apply protoplast cultures to the aforementioned approaches in this research, we established the protoplast-to-plant system in Tartary buckwheat.

**Results:**

In this work, cellulase and pectinase activity enabled protoplast isolation from non-morphogenic and morphogenic callus (MC), reaching, on average, 2.3 × 10^6^ protoplasts per g of fresh weight. However, to release protoplasts from hypocotyls, the key step was the application of driselase in the enzyme mixture. We showed that colony formation could be induced after protoplast embedding in agarose compared to the alginate matrix. Protoplasts cultured in a medium based on Kao and Michayluk supplemented with phytosulfokine (PSK) rebuilt cell walls, underwent repeated mitotic division, formed aggregates, which consequently led to callus formation. Plating efficiency, expressing the number of cell aggregate formed, in 10-day-old protoplast cultures varied from 14% for morphogenic callus to 30% for hypocotyls used as a protoplast source. However plant regeneration via somatic embryogenesis and organogenesis occurred only during the cultivation of MC-derived protoplasts.

**Conclusions:**

This study demonstrated that the applied protoplast isolation approach facilitated the recovery of viable protoplasts. Moreover, the embedding of protoplasts in an agarose matrix and supplementation of a culture medium with PSK effectively stimulated cell division and further development of Tartary buckwheat protoplast cultures along with the plant regeneration. Together, these results provide the first evidence of developing a protoplast-to-plant system from the MC of *Fagopyrum tataricum* used as source material. These findings suggest that Tartary buckwheat’s protoplast cultures have potential implications for the species’ somatic hybridization and genetic improvement.

## Background

*Fagopyrum tataricum* (L.) Gaertn., known as Tartary buckwheat, is one of the two most widely cultivated buckwheat species belonging to the family *Polygonaceae*. This self-pollinating, annual and dicotyledonous crop is grown in difficult climatic conditions, mainly in the mountain regions of southwest China [1, 2]. It is an excellent natural source of biologically active substances containing many flavonoids and phenolic compounds, especially rutin, quercetin and C-glycosylflavones, which has been used primarily in herbal medicine and the pharmaceutical industry [3, 4]. Flavonoid compounds improve the elasticity of the veins and support the circulatory system, while rutin is used in treating postoperative scars or body burns due to X-rays radiation [5]. Moreover, buckwheat is a rich source of starch, high-quality proteins, antioxidants, dietary fibre, vitamins and trace elements [6, 7]. Likely to common buckwheat (*Fagopyrum esculentum* L.) Tartary buckwheat is a plant with a health-promoting effect on the human body [8, 9]. In addition, it was shown that in plants, rutin enhances the defence system against environmental stress factors like UV light, low temperature, and desiccation [10]. Likewise, the high concentration of rutin protects buckwheat plants against insect pests [11] and has an effect on deterring animals [12]. The relatively good fatty acid composition, high dietary fibre content, and high vitamin B level make this plant an excellent food material with potential medicinal and pharmaceutical applications [13]. The nutraceutical properties of Tartary and common buckwheat include anti-oxidant, anti-ageing, anti-neoplastic properties, and cardio-protective and hepato-protective properties [4].

So far, in vitro culture systems for callus induction, plant regeneration, and the synthesis of phenolic compounds have been studied for buckwheat [14]. Protoplast-based procedures are one of the new plant breeding technologies that may be promising for buckwheat crop improvement [15]. Nonetheless, the possibility of protoplast regeneration into plants is fundamental in the successful application of somatic hybridisation or protoplast transformation [16] for transferring significant agronomical traits (i.e. tolerance to biotic/abiotic stresses and higher content of beneficial compounds) from wild *Fagopyrum* species [17]. Additionally, the buckwheat protoplast-based techniques may help obtain gene-edited plants with improved agronomical features by applying protoplast transfection. Nowadays, applying biotechnology tools to Tartary buckwheat may attract scientists due to it producing metabolites essential for preserving human health, creating genetically transformed plants and generating somatic hybrids [2, 16] as well in developmental biology research to the subcellular localisation of proteins and the assessment of gene activity [18].

Using protoplast cultures as a routine research tool requires the examination of different cultivars, ecotypes, and plant tissues to choose those with the best developmental and regenerative response in protoplast cultures [19–21]. The next crucial step is selecting an appropriate protoplast culture technique among cultures in liquid, semi-solid or solid medium with agar, agarose or alginate. Additionally, protoplast development can be ensured by applying additional supplements, such as peptide growth factors, polyamines or inhibitors of phenolics compounds. An excellent example of peptide growth factors application is PSK - a sulphated pentapeptide that promotes cell growth and proliferation [22], enhances the growth of callus [23], roots [24], shoots [25], and buds formation [26] and can improve somatic embryogenesis [27, 28]. Other compounds, such as polyamines, impact the maintenance of protoplast viability, increase mitotic activity and shoot regeneration and decrease oxidative stress [29]. The oxidation of phenolics in tissue culture harms the growth of tissues in in vitro conditions and leads to the browning of tissues and the growth medium. As a result, it reduces tissue growth, decreases regeneration rates and leads to cell culture necrosis [30]. Therefore, to reduce tissue browning, some compounds can be applied. Polyvinylpyrrolidone (PVP) is used to absorb phenolics released during protoplast cultures [31–33] or the propagation of woody plant species [34]. Another is 2-aminoindane-2-phosphonic acid (AIP), a specific competitive phenylalanine ammonia-lyase (PAL) inhibitor [30, 35, 36]. It should be noted that the application of AIP reduced flavonoid content and increased protoplast isolation frequency, effected on cell wall reconstruction, cell division, and decreased browning of suspension and callus culture of the *Ulmus americana* L [30, 36]. An alternative approach is to use some antioxidants. Ascorbic acid, citric acid and activated charcoal eliminate phenolics and other substances secreted into the culture medium by explants [32, 37–40]. The addition of activated charcoal to the protoplast culture medium improved colony and microcalli formation in chrysanthemum-derived protoplast cultures [39] and overcame the problem of cell browning during protoplast cultures of *Eustoma grandiflorum* [38], *Vitis vinifera* L [41]. or *Solanum tuberosum* L [40].

The literature data concerning protoplast cultures of the buckwheat species are limited. So far, only one successful plant regeneration from hypocotyl-derived protoplasts of common buckwheat has been published [42]. In the case of Tartary buckwheat, Lachman and Adachi [43] reported callus formation in hypocotyl-derived protoplast cultures. Therefore, the main objective of this study was to (1) identify some factors promoting protoplast development and (2) develop a protoplast-based system for plant regeneration in Tartary buckwheat.

## Results

**Fig. 1:**
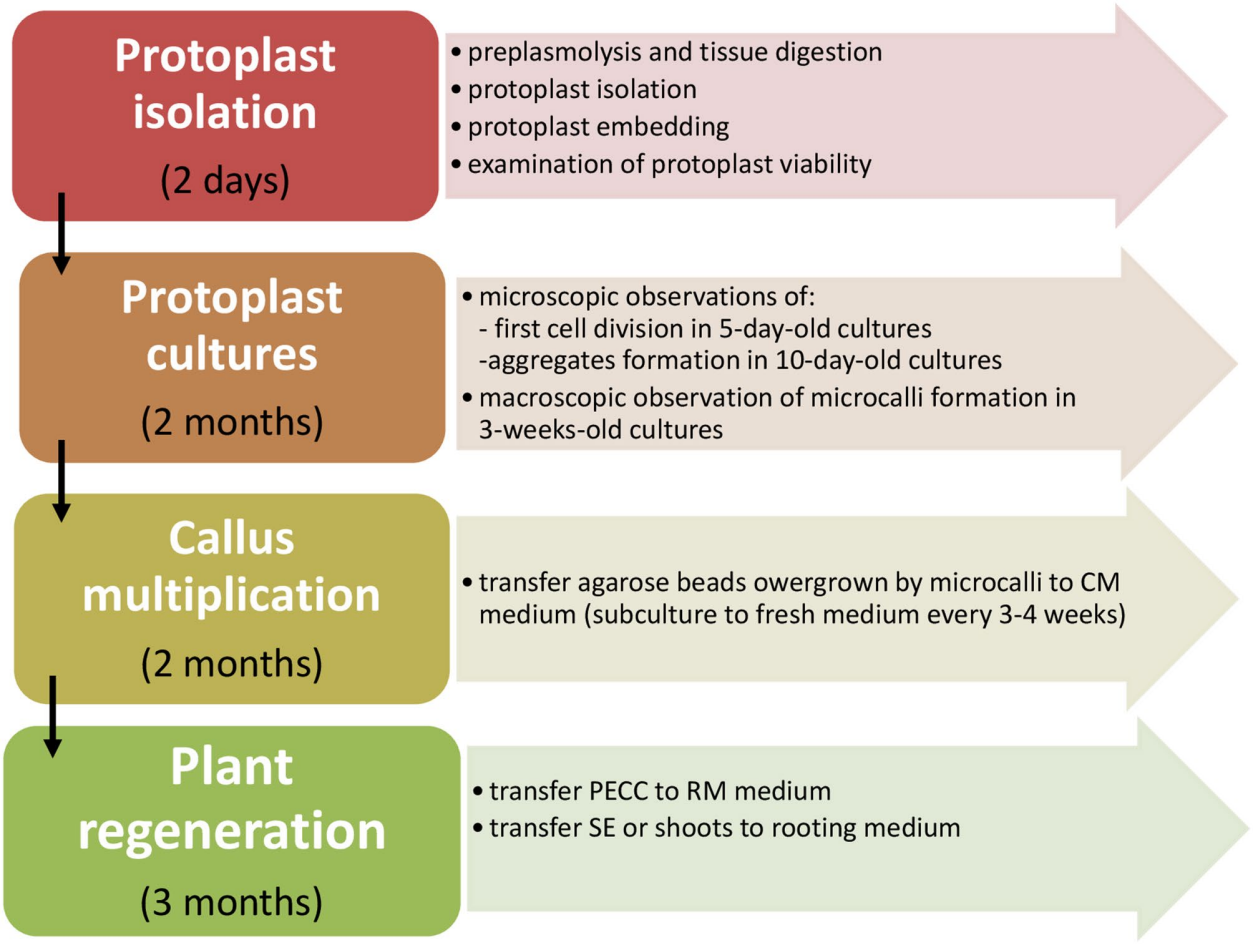
Flow chart illustrating a step-by-step approach for plant regeneration via protoplast cultures of *Fagopyrum tataricum*. Details are described in the method section. *CM* callus multiplication medium; *PECC* pro-embryogenic cell complexes; *RM* regeneration medium; *SE* somatic embryos

Comprehensive protoplast cultures and plant regeneration were carried out as presented in Fig. 1.

### Morphology of callus used as protoplast source

**Fig. 2:**
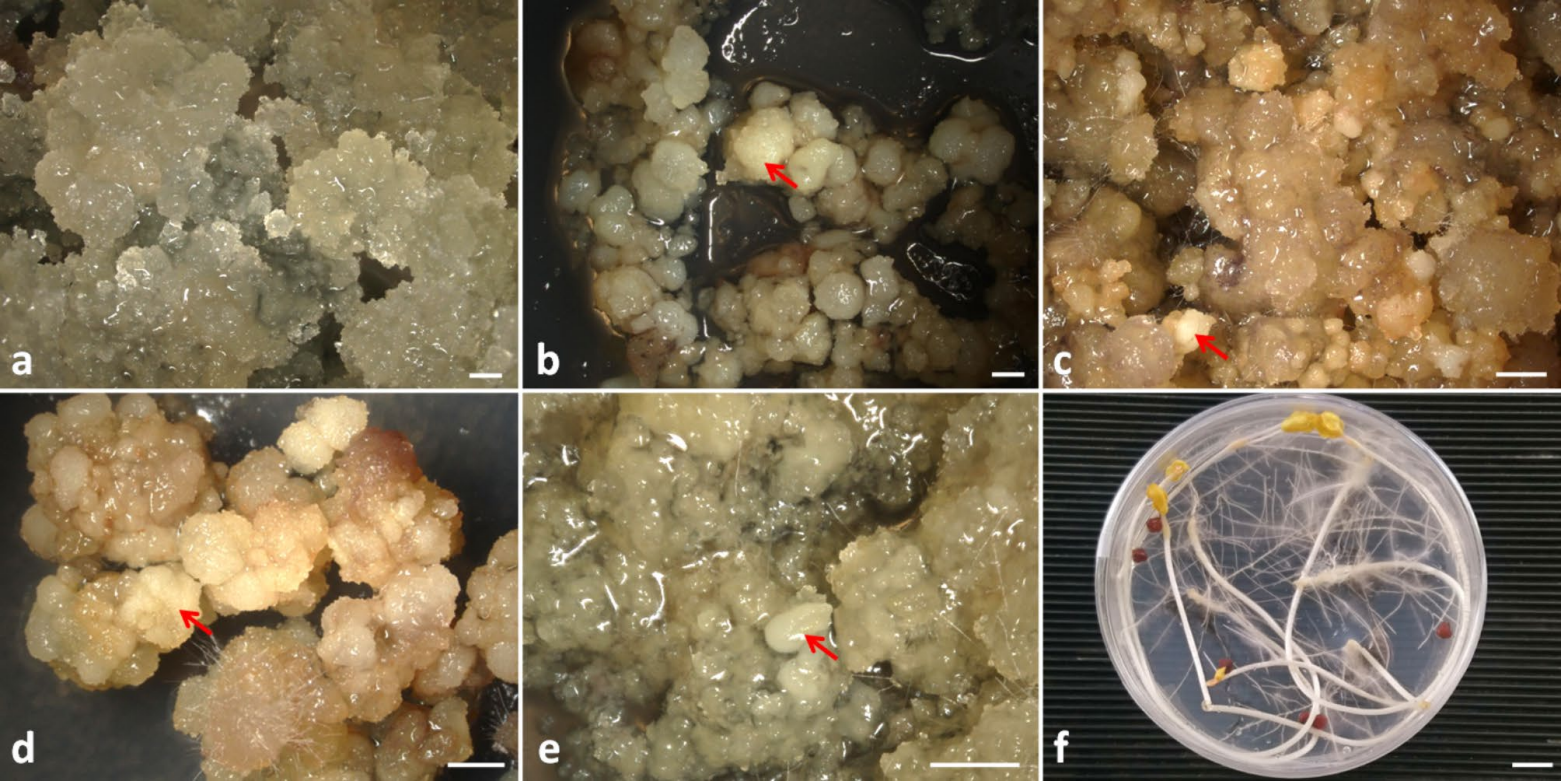
Donor callus (**a-e**) and 10-day-old hypocotyls (**f**) of *Fagopyrum tataricum* used as source material for protoplast isolation. Morphology of 2-week-old callus lines: (**a**) non-morphogenic (NC) and morphogenic (MC) callus: (**b**) MC1, (**c**) MC2, (**d**) MC4, (**e**) NL2018. Arrows show proembryogenic cell complexes (PECCs) of MC. Scale bars: 1 mm (**a-e**), 1 cm (**f**)

Protoplasts were isolated from one line of the non-morphogenic callus (NC, Fig. 2a) and four lines of the morphogenic callus (MC1, MC2, MC4, NL2018, Fig. 2b-e) of *Fagopyrum tataricum.* The 7-year-old NC line was characterised by a fragile structure and rapid growth and was formed exclusively from parenchymatous-type cells, which emerged on the surface of the MC1 line after several years of culture. On the other hand, the MC lines were varied in age; they were 10-, 4- and 2-year-old for MC1 and MC2; NL2018; MC4, respectively. They consisted of proembryogenic cell complexes (PECCs) and a ‘soft’ callus that appears during the cyclical disintegration of PECCs. PECCs are white structures (nodules) on the callus surface that appear one week after transfer to fresh medium. Therefore, the protoplasts were isolated from a 1-2-week-old callus, counting from the previous passage. The three lines of MC were different in the size of PECCs. The MC1, MC2 and MC4 lines had similar PECCs (Fig. 2b-d, red arrows), in contrast to the line NL2018, characterised by very small PECCs (Fig. 2e, red arrow). Probably the softer structure of the line NL2018 effect the protoplast quality. The cells of NL2018 were not destroyed during protoplast cultures compared to the rest of the morphogenic lines.

### Yield and viability of released protoplasts

**Fig. 3:**
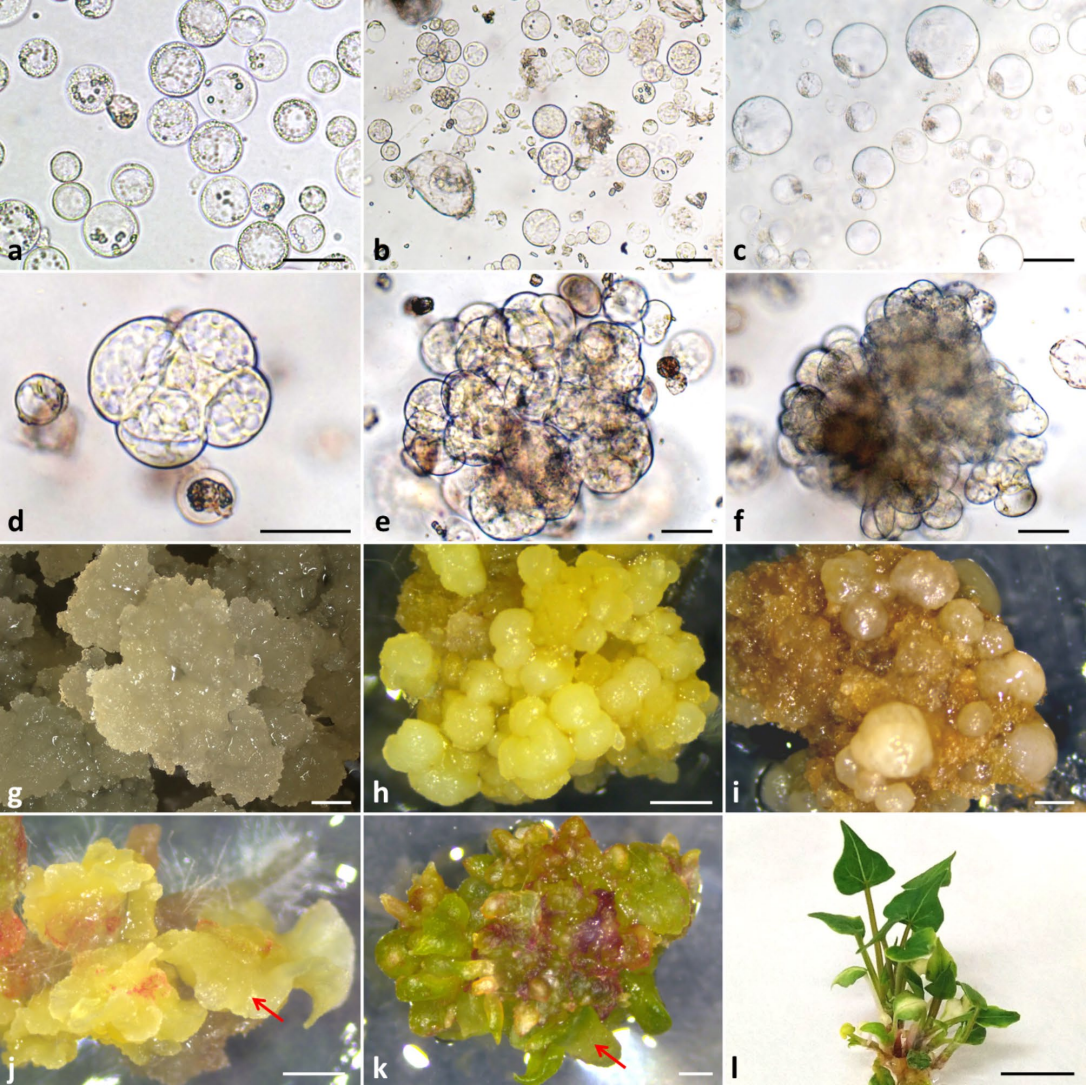
Plant regeneration in protoplast cultures of *Fagopyrum tataricum*. Freshly isolated protoplasts from (**a**) non-morphogenic callus (NC), (**b**) morphogenic callus (MC) and (**c**) hypocotyls; multicellular aggregate in 8- (**d**), 10- (**e**), 20- (**f**) day-old protoplast cultures originating from MC; callus obtained from NC- (**g**), MC- (**h**) and hypocotyl- (**i**) derived protoplast cultures four months after protoplast isolation; subsequent stages of plant regeneration via somatic embryogenesis (**j**) and organogenesis (**k**) with - arrow indicating somatic embryo and shoot, respectively (after one month of regeneration); (**l**) plant of Tartary buckwheat regenerated from MC-derived protoplast cultures (after two month of regeneration). Scale bars: 50 μm (**a-f**), 1 mm (**g-k**), 1.5 cm (**l**)

Spherical protoplasts (Fig. 3a-c) were successfully isolated from NC, MC and hypocotyls (Fig. 2), and used as source material. The mean yield of NC protoplasts (0.43 ± 0.09 × 10^6^) was six to nine times lower compared to MC protoplasts (Table 1). The highest protoplast yield from MC was noted for line NL2018 (3.93 ± 0.09 × 10^6^), while the lowest was for the MC1 line (2.30 ± 0.38 × 10^6^).

Different concentrations of driselase (a mix of several cell wall-degrading enzymes) to the enzyme mixture were applied to release protoplasts from the hypocotyl tissue and improve protoplast yield. The efficiency of protoplast yield reached, on average, 0.51 × 10^6^ cells per g of tissue (Table 2). The mean number of released protoplasts varied from 0.39 × 10^6^ after applying 0.25% driselase to 0.71 × 10^6^ for 0.1% of driselase. However, differences observed in protoplast yield after applying different concentrations of driselase were insignificant. The average yield of hypocotyl-derived protoplasts was five-fold lower than from MC sources (*P* ≤ 0.01).

Callus and hypocotyl-derived protoplasts, just after embedding in agarose beads, showed different viability as determined by fluorescein diacetate (FDA) staining (Tables 1 and 2). The viability of callus-derived protoplasts varied from 55% for NC to 78% for line NL2018; however, the observed differences were not significant (Table 1). Hypocotyl-derived protoplasts showed a different level of protoplast viability, depending on the driselase concentration during the maceration stage. The highest viability of hypocotyl protoplasts (81%) was obtained when digestion was performed using 0.25% driselase in the enzyme mixture.

**Table 1:** Isolation efficiency and viability of *Fagopyrum tataricum* callus-derived protoplasts

Protoplast source	Callus	Protoplast yield (× 10^6^/g FW)		Protoplast viability (%)	
	line	n	Mean ± SE	n	Mean ± SE
Non-morphogenic callus	NC	3	0.43 ± 0.09^c^	2	54.50 ± 5.50^a^
Morphogenic callus	MC1	3	2.30 ± 0.38^a^	3	66.67 ± 7.67^a^
	MC2	2	2.44 ± 0.46^ab^	2	68.75 ± 2.75^a^
	MC4	2	2.40 ± 0.50^ab^	2	64.00 ± 8.00^a^
	NL2018	3	3.93 ± 0.09^b^	2	77.93 ± 4.56^a^
Mean/Total		13	2.28 ± 0.36	11	67.36 ± 3.23

*FW* fresh weight; *n* number of independent protoplast isolations. Means followed by the same letters within a column were not significantly different at *P* ≤ 0.05.

**Table 2:** Effect of driselase concentration on yield and viability of protoplasts originating from *Fagopyrum tataricum* hypocotyls

Driselase	Protoplast yield (× 10^6^/g FW)		Protoplast viability (%)	
concentration (%)	n	Mean ± SE	n	Mean ± SE
0.10	2	0.71 ± 0.06^a^	2	72.00 ± 0^ab^
0.15	2	0.43 ± 0.18^a^	2	63.50 ± 0.50^a^
0.25	2	0.39 ± 0.01^a^	2	81.50 ± 3.50^b^
Mean/Total	6	0.51 ± 0.08	6	72.33 ± 3.41

*FW* fresh weight; *n* number of independent protoplast isolations. Means followed by the same letters within a column were not significantly different at *P* ≤ 0.05.

### Development of protoplast cultures

**Fig. 4:**
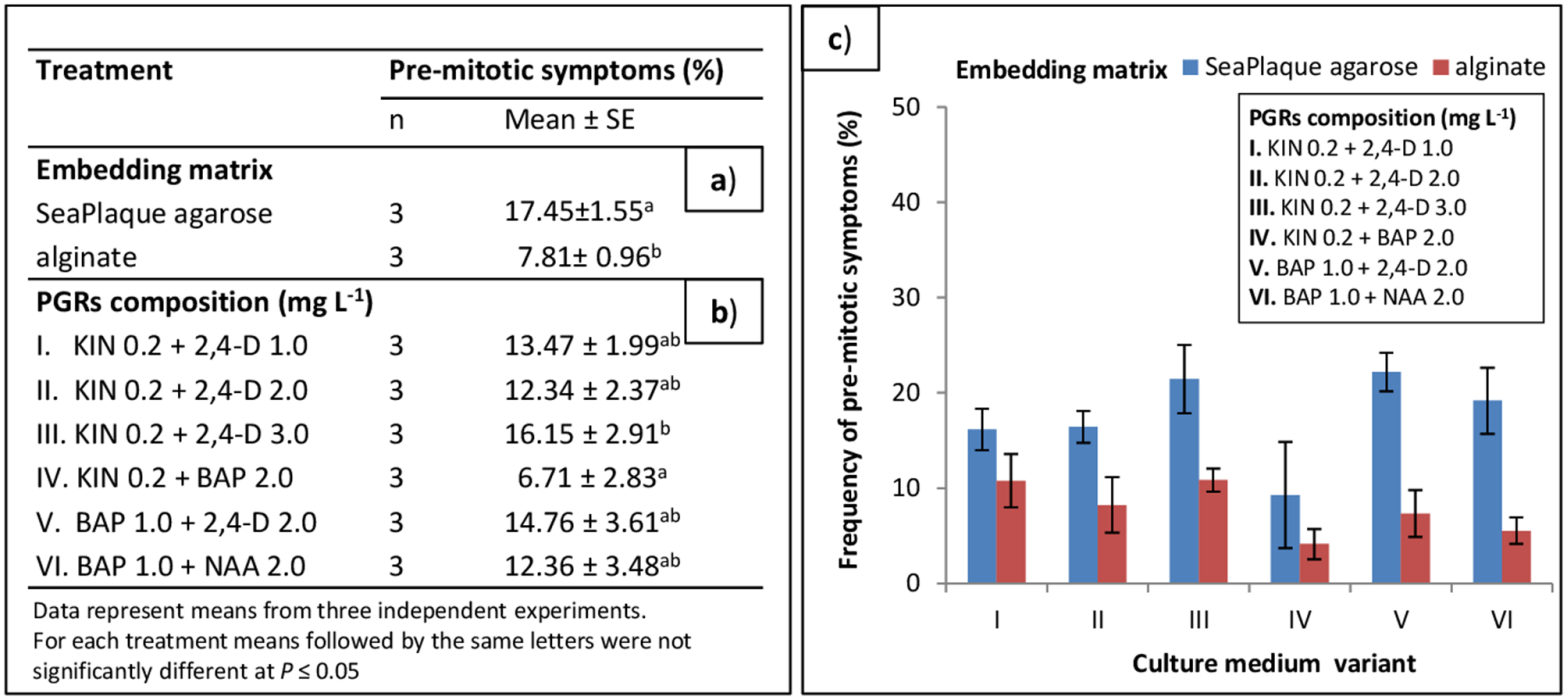
Frequency of pre-mitotic symptoms in 10-day-old protoplast cultures originating from non-morphogenic callus of *Fagopyrum tataricum.* Effect of (**a**) embedding matrix, (**b**) plant growth regulators (PGRs) and (**c**) both treatments on culture development. *BAP* = 6-benzylaminopurine; *2,4-D* = 2,4 dichlorophenoxy acetic acid; *KIN* = kinetin; *NAA* = α-naphthalene acetic acid; *n* = number of independent protoplast isolations; *SE* = standard error. In chart bars represent means of three independent experiments ± SE

In preliminary experiments performed on NC protoplasts, (1) type of protoplast embedding matrix and (2) plant growth regulators (PGRs) composition in culture medium were examined. In 10-day-old cultures, positive symptom characteristics for the pre-mitotic period were observed, including: (1) cells enlargement in size, (2) change of the cell shape from spherical to oval, which was the morphological evidence of cell wall reconstruction and (3) reorganisation of the cytoplasm and cell organelles. Out of two applied embedding systems, immobilisation of protoplasts in SeaPlaque agarose better affected cell development. On average, twice as many pre-mitotic symptoms were observed in comparison to the alginate embedding system (Fig. 4a). Auxins and cytokinins used in various concentrations in culture medium also influenced the occurrence of pre-mitotic symptoms (Fig. 4b). The highest number (16%) of cells with positive symptoms was observed in culture variant medium III (supplemented with 0.2 mg L^− 1^ kinetin (KIN) and 3.0 mg L^− 1^ 2,4-dichlorophenoxy acetic acid (2,4-D)), while the lowest (6.7%) was observed in medium IV (supplemented with 0.2 mg L^− 1^ KIN and 2.0 mg L^− 1^ 6-benzylaminopurine (BAP)), independent of the protoplast embedding system (Fig. 4c). In culture media variants I, II, V and VI the frequency of pre-mitotic symptoms was similar and reached, on average, 13% (Fig. 4b). Based on these results, in further experiments, protoplasts were embedded in agarose.

**Fig. 5:**
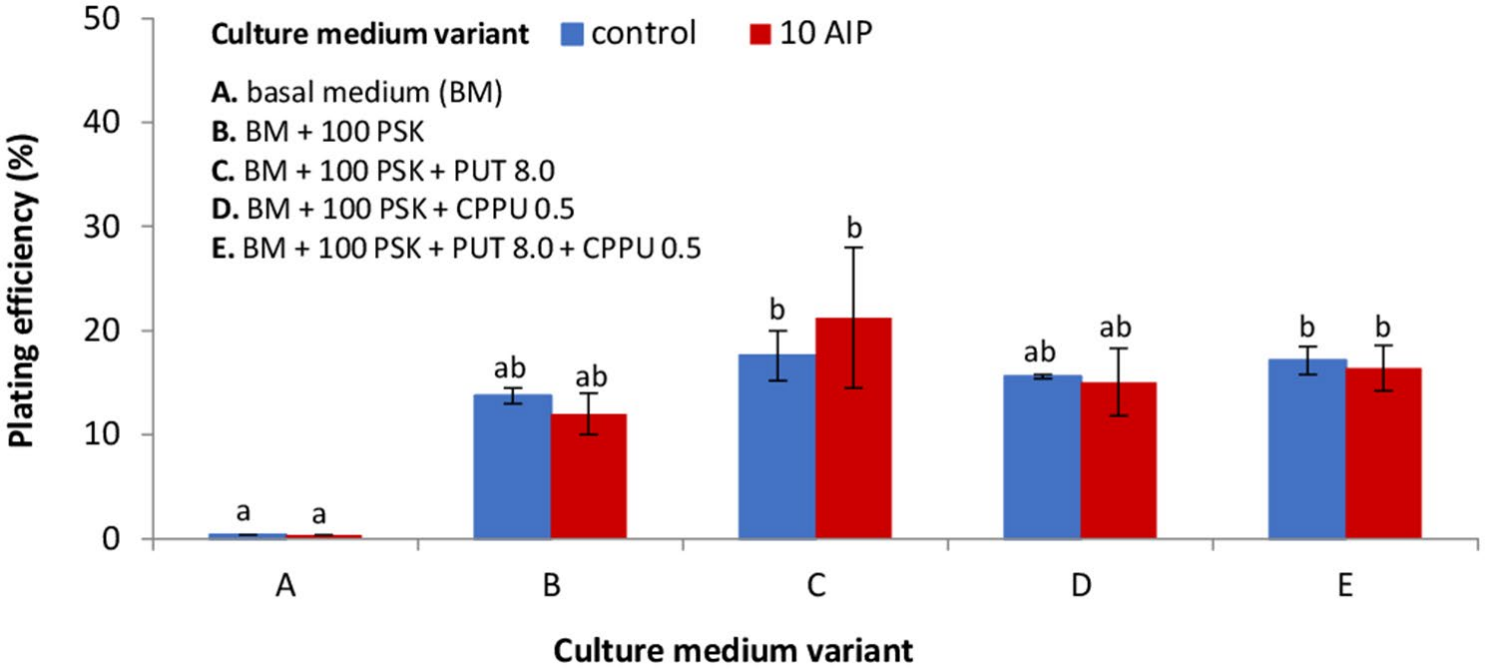
Effect of plant growth regulators (PGRs) and AIP on plating efficiency in 10-day-old protoplast cultures originating from morphogenic callus (line NL2018) of *Fagopyrum tataricum.* PGRs composition in *BM* for protoplast cultures = 1.0 mg L^− 1^ BAP (6-benzylaminopurine) + 2.0 mg L^− 1^NAA (α-naphthalene acetic acid); *AIP =* 10 µM 2-aminoindane-2-phosphonic acid; *100 PSK* = 100 nM phytosulfokine; *CPPU 0.5* = 0.5 mg L^− 1^ N-(2-chloro-4-pyridyl)-N’-phenylurea; *PUT 8.0* = 8 mg L^− 1^ putrescine. Bars represent means from two independent experiments ± SE (standard error). Means marked with the same letters were not significantly different at *P ≤ 0.05*

**Fig. 6:**
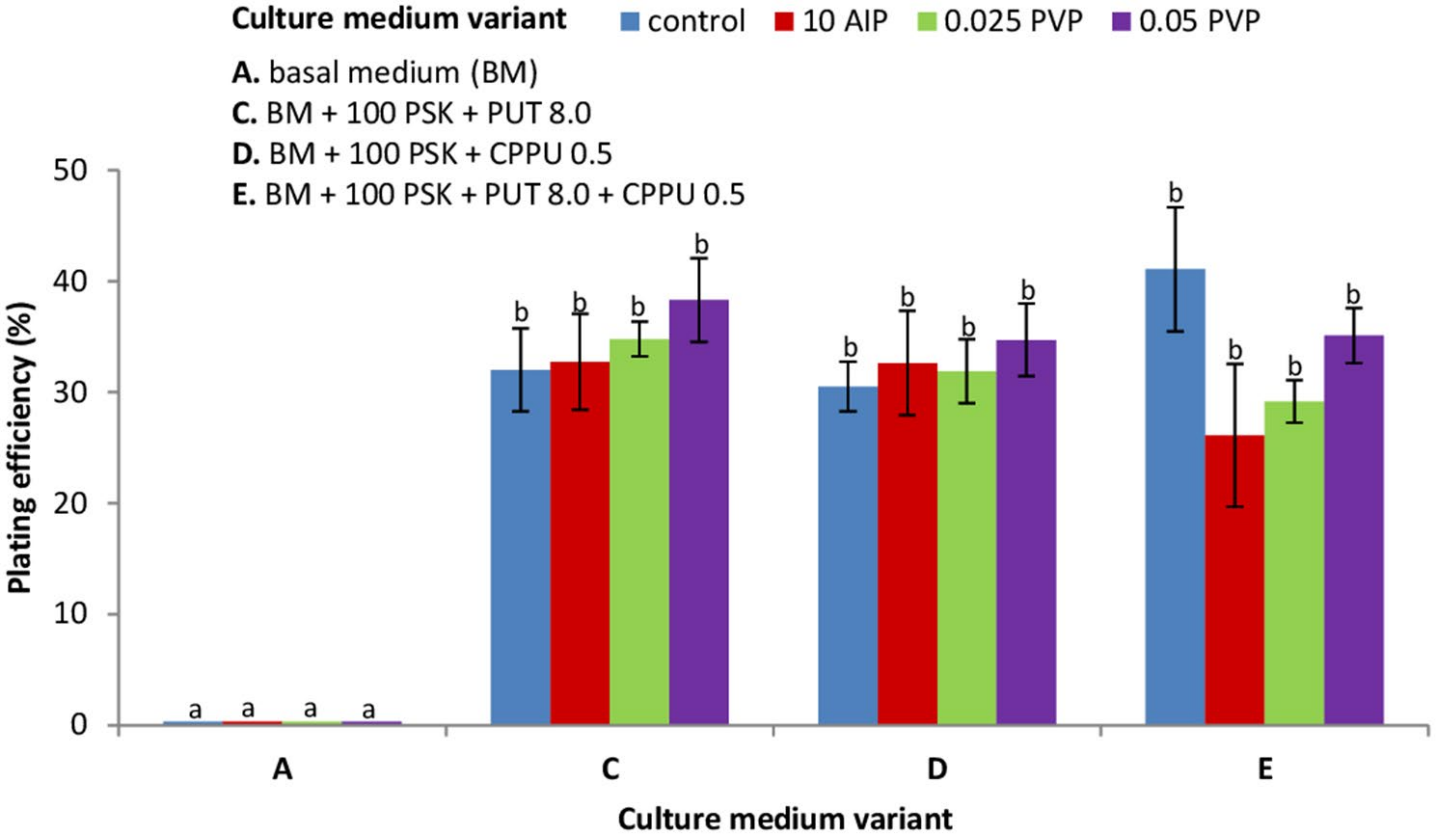
Effect of plant growth regulators (PGRs) and compounds inhibiting (AIP) or absorbing (PVP) phenolics on plating efficiency in 10-day-old protoplast cultures originating from hypocotyls of *Fagopyrum tataricum*. PGRs composition in *BM* for protoplast cultures = 1.0 mg L^− 1^ BAP (6-benzylaminopurine) + 2.0 mg L^− 1^NAA (α-naphthalene acetic acid); *100 PSK* = 100 nM phytosulfokine; *CPPU 0.5* = 0.5 mg L^− 1^ N-(2-chloro-4-pyridyl)-N’-phenylurea; *PUT 8.0* = 8 mg L^− 1^ putrescine; *10 AIP =* 10 µM 2-aminoindane-2-phosphonic acid; *0.025, 0.05 PVP* = 0.025% or 0.05% polyvinylpyrrolidone, respectively. Bars represent means from two to five independent experiments ± SE (standard error). Means marked with the same letters were not significantly different at *P ≤ 0.05*

The MC1 line was used as a protoplast source in the preliminary experiments with morphogenic callus. Protoplasts embedded in agarose beads were cultured in the same six culture variants media as applied to NC-derived protoplasts (Fig. 4b). In 10-day-old cultures, mainly negative symptoms such as plasmolysis, broken cells or cells without developmental features were observed. However, in 2-month-old cultures in medium variant VI the microcalli was formed. Based on that observation, the medium variant VI was applied to the following protoplast cultures and named as basal medium (BM) for protoplast cultures. Among tested MC lines, only the NL2018 line revealed the ability to undergo cell divisions in protoplast cultures. Supplementation of the BM with PSK showed a beneficial effect on the mitotic activity of MC- and hypocotyls protoplast-derived cells (Figs. 5 and 6). Although first mitotic divisions were occasionally observed in the 5-day-old protoplast cultures of MC and hypocotyls, multicellular aggregates were already formed in 8-day-old cultures (Fig. 3d). As determined under the microscope, cells rich with dense cytoplasm in the aggregates were tightly packed, suggesting their embryogenic competence (Fig. 3e, f).

In 10-day-old protoplast cultures, the plating efficiency demonstrated by the number of cell aggregates formed was determined. For MC-derived protoplast cultures, this parameter ranged from 14–18% (Fig. 5) in control medium variants and from 12–21% for variants supplemented with AIP (Fig. 5). Nevertheless, differences in protoplast efficiency after the application of AIP were statistically insignificant. In culture two variant media (C and E) supplemented with PSK, putrescine (PUT) and N-(2-chloro-4-pyridyl)-N’-phenyl urea (CPPU), the highest number of cell aggregates (from 16 to 21%) was observed (Fig. 5).

In 10-day-old hypocotyl protoplast cultures, the number of cell aggregates varied, depending on the culture medium variant, from 25 to 41%, however, observed differences were statistically insignificant (Fig. 6). AIP and PVP applied additionally to the culture media to reduce the accumulation of phenolics and thus avoid culture browning did not influence the positive development of the culture. About twice the higher level of plating efficiency (33%) was observed in hypocotyl protoplast cultures compared to the MC protoplast cultures (15%).

Independently on the protoplast source, multicellular aggregates continued to grow and become macroscopically visible after approximately three weeks of the culture. In the eighth week of culture, microcalli overgrew the agarose beads with different intensity, depending on the protoplast source. Medium development of microcalli was noted for NC- and MC-derived protoplast cultures. In the case of hypocotyl-derived protoplast cultures, the agarose beads were overgrown completely by microcalli. For NC-derived protoplast cultures the microcalli were observed for all medium variants except variant IV. For MC- and hypocotyl-derived protoplast cultures, microcalli developed regardless of the culture medium variant. Additionally, it was observed that the application of PVP to the culture reduced both the amount of floating metabolites in the protoplast medium and the browning of microcalli.

### Histological observations of protoplast-derived callus

**Fig. 7:**
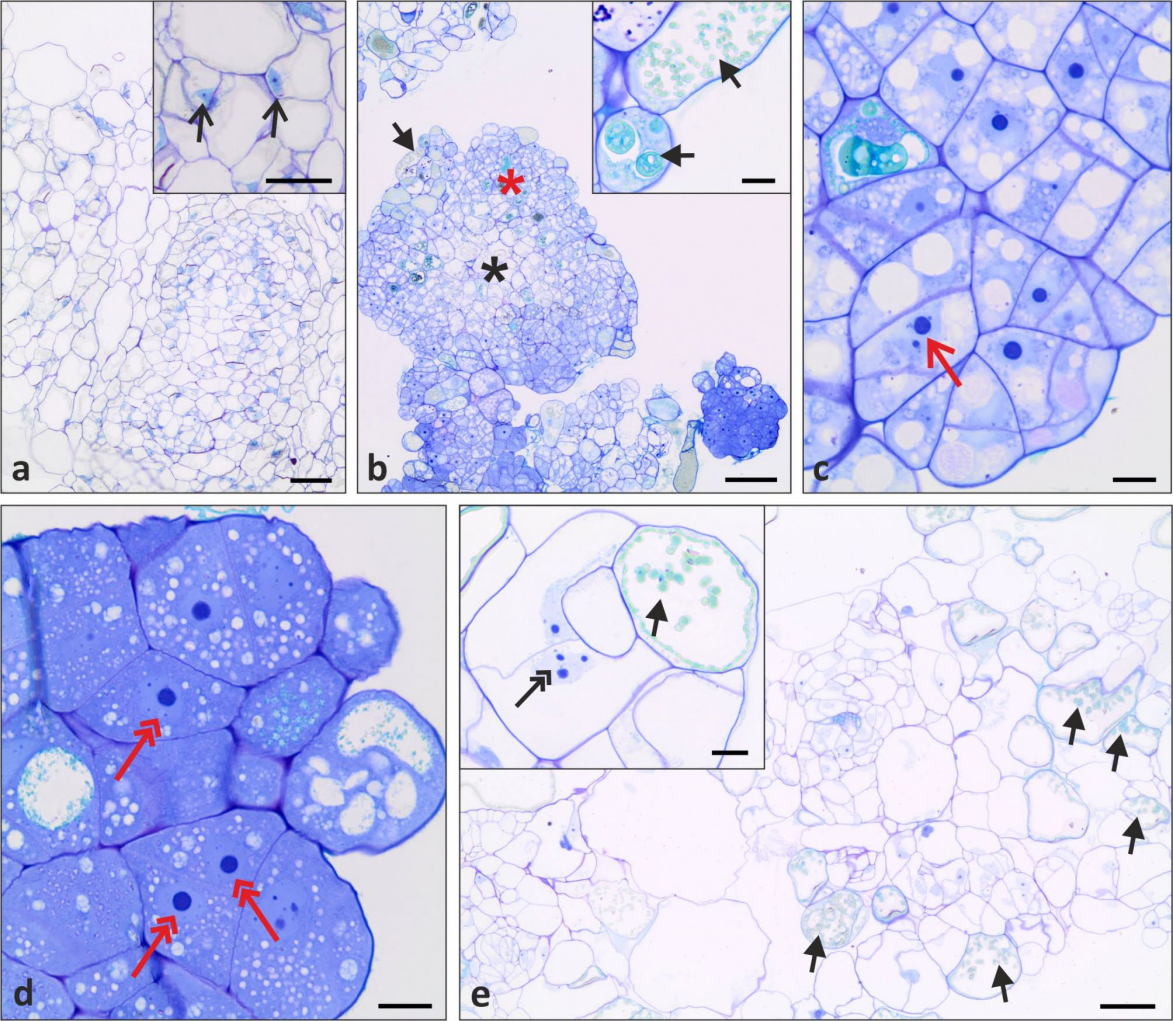
Histological sections of protoplast-derived callus originating from: (**a**) non-morphogenic callus (NC), (**b-d**) morphogenic callus (MC; line NL2018) and (**e**) hypocotyls of *Fagopyrum tataricum*. Protoplast cultures from NC consisted of parenchymatous cells (**a**) with large vacuole and the nucleus in the periphery of the cell (**a** inset). Callus developed from MC-derived protoplasts (**b-d**) had morphogenic potential and the PECCs were observed (**b**). This callus consisted of phenolic-containing cells (**b** inset), meristematic cells (**c**) and embryogenic cells (**d**). Callus from hypocotyl-derived protoplast cultures was made of parenchymatous cells (**e**) and contained some cells with phenolic compounds (**e** inset) and some cells with nucleus with two or three nucleoli (**e** inset). Black open arrows show nucleus in the periphery of parenchymatous cells; black asterisk indicates parenchymatous cells of PECCs; red asterisk indicates meristematic cells of PECCs; black arrows indicate phenolic compounds; red open arrow shows nucleus with two nucleoli in meristematic cell; red double arrows indicate nucleus with large nucleoli in embryogenic cells; black double arrow shows nucleus with three nucleoli in parenchymatous cells. Scale bars: 10 μm (**b** inset, **c**, **d**, **e** inset), 50 μm (**a**, **a** inset, **e**), 100 μm (**b**)

Histological observations revealed that callus developed from NC-derived protoplasts was composed of thin-walled parenchymatous cells, some of which were loosely arranged (Fig. 7a). These cells varied in sizes, with a large vacuole and an irregular nucleus on the periphery of the cell protoplast (Fig. 7a inset). In the case of microcalli from morphogenic callus-derived protoplasts (line NL2018), histological analysis showed heterogenous callus with PECCs present, and thus several types of cells can be distinguished (Fig. 7b-d). The calli’s surface noted some phenolic-containing cells (PCC) that had a large central vacuole in which phenolic compounds were accumulated (Fig. 7b and b inset, black arrows). Subsurficial tissue was composed of meristematic cells (Fig. 7b, red asterisk; Fig. 7c) that were characterised by the presence of several vacuoles, dense cytoplasm and round-shape nucleus with visible one or two nucleoli (Fig. 7c, red open arrow). The parenchymatous cells were present in the centre of PECCs (Fig. 7b, black asterisk). Histological observations confirmed the presence of embryogenic cells characterised by very dense cytoplasm, numerous small vacuoles and a large, round nucleus with one big nucleoli (Fig. 7d, red double arrows). Microcalli obtained from hypocotyl-derived protoplasts consisted of a mass of loosely arranged thin-walled parenchymatous cells (Fig. 7e). The vacuoles occupied almost the entire volume of the cells. As a result, the nucleus was located peripherally in the vicinity of the cell membrane (Fig. 7e inset). The nucleus was irregular in shape, and one to three nucleoli were observed (Fig. 7e inset, black open arrow). In some cells, the presence of phenolic compounds in the vacuole was detected (greenish colour after Toluidine Blue O staining; Fig. 7e and e inset, black arrows).

### Plant regeneration from protoplast-derived tissue

Two-month-old protoplast-derived callus doubled its mass within the next two months on callus multiplication medium additionally enriched with PSK. Friable NC (Fig. 3g), soft callus with PECCs (Fig. 3h), and non-embryogenic callus (Fig. 3i) were observed in the cultures originating from NC, MC and hypocotyl protoplasts, respectively. After one month on the regeneration medium, the calli originating from MC protoplasts formed somatic embryos (Fig. 3j) and shoots (Fig. 3k). Finally, after about three months, plants without morphological abnormalities were produced (Fig. 3l).

## Discussion

Plant protoplasts can dedifferentiate, re-enter the cell cycle, undergo repeated mitotic divisions, and develop into fertile plants [44, 45]. The protoplast technique has great potential for studying developmental biology [46], responses to stress conditions [25], in vitro selection or the production of useful secondary metabolites [47]. Especially the protoplast fusion and subsequent in vitro plant regeneration, as a tool of somatic hybridisation, offer opportunities for transferring entire genomes from one plant into another regardless of the interspecific crossing barriers [44].

Several source materials with different genotypes, cultivars, ages, and growth conditions of the source tissue are used by researchers for protoplast isolation [15]. In this research, protoplasts were isolated from callus (NC and MC) and hypocotyls to select material characterised by high regeneration capacity in protoplast cultures. In our study, a satisfactory number of protoplasts was achieved, reaching more than 2 × 10^6^ protoplasts per g of callus and around 0.5 × 10^6^ protoplasts per g of hypocotyls tissue. Similarly to our observations, a reduced number of hypocotyl-derived protoplasts in contrast to other source materials (e.g. leaves) was observed in studies on *Brassica oleracea* [48] and *Daucus carota* [21].

In order to improve the production of protoplasts from hypocotyl tissue, we applied driselase in the enzyme mixture. According to Thibault and Rouau [49], driselase is especially active towards carboxymethyl cellulose and hemicelluloses (xylan and laminarin). Those authors revealed that the application of driselase resulted in almost completely degraded polysaccharides (rhamnose, arabinose, galactose and glucuronic acid) in fibres from sugar beet pulp [49]. According to Lachmann and Adachi [43], it was possible to release protoplasts from 7-day-old hypocotyls of Tartary buckwheat without driselase. It seems that the genotype and the hypocotyl age might significantly influence the efficiency of protoplast isolation. Nevertheless, we noted satisfactory protoplast yield from the hypocotyls after the application of driselase. The activity of driselase may suggest that hypocotyl cell walls contain hemicelluloses such as laminarin and xylan, and therefore applying enzyme solution without driselase was unsuccessful. There is no literature data to confirm this suggestion, and this hypothesis will need further biochemical verification. Several authors have demonstrated that the addition of the driselase to the enzymatic mixture increased the protoplast yield isolated from *Kalanchoe blossfeldiana* [50], *Spathiphyllum wallisii, Anthurium scherzerianum* [51] and *Brassica oleracea* [52].

Different protoplast culture systems can be used, however, the immobilisation of protoplasts in a semi-solid medium ensures the physical separation of cells, decreases the production of polyphenols and prevents necrosis in the protoplast cultures [45, 53]. Interestingly, alginate is a common use alternative to agar or agarose. For *Daucus carota* [21, 54], *Brassica oleracea* [48] and *Beta vulgaris* [55], an increase in division frequency after protoplasts immobilisation in alginate was shown. In Tartary buckwheat, we did not observe such a positive effect of the alginate matrix on callus- and hypocotyl-derived protoplast cultures. However, the results of our study strongly demonstrated that the immobilisation of Tartary buckwheat protoplasts in agarose beads positively impacts their development. According to Brodelius and Nilsson [56], the production of secondary products from precursors and carbon sources was lower by the immobilised cells in agarose than for those embedded in alginate. Thus, we presume that immobilising Tartary buckwheat protoplasts in agarose might reduce the harmful secondary metabolites produced during protoplast cultures. Additionally, the applied SeaPlaque agarose is characterised by the reduction of helix structure and enables rapid delivery of gases and substances (hormones, signalling molecules, metabolites) to the embedded cells [57, 58]. Moreover, Shoichet et al. [59] demonstrated that the gel strength of cell-containing agarose, in contrast to alginate, is lower, which is connected with a reduction of cross-links between polymer chains of agarose. In the context of protoplast cultures, it makes it possible to increase in the space allowing the diffusion of the substances that were mentioned above. After applying the low melting point bead technique, similar results were achieved in *Ulmus americana* protoplast cultures [60]. Also Pan et al. [61] reported that agarose was essential for cell division and colony formation for *Artemisia judaica* while alginate better affected the development of *Echinops spinosissimus* protoplasts.

Protoplast culture media, especially PGRs, are necessary for persistent mitotic divisions of protoplast-derived cells, aggregates formation, and their differentiation into plants [53]. According to Lachmann and Adachi [43], hypocotyl-derived protoplasts of Tartary buckwheat initiated cell division after three to five days after initiation of the culture. They formed cell aggregates in the medium enriched with BAP and naphthaleneacetic acid (NAA). In another research on common buckwheat protoplasts, Adachi et al. [42], after the application of different combinations of hormones, demonstrated the best response of protoplast development in a medium enriched with BAP and NAA. Our study demonstrated that only after applying PSK to BM medium supplemented with BAP and NAA, the first cell divisions took place in five-day-old cultures and the following development of protoplast cultures was observed. Thus, it seems that these hormones can be universal and used for both Tartary and common buckwheat.

A common way to support protoplast division and microcalli formation involves the application of additional supplements, such as peptide growth factors, polyamines, and compounds which can absorb or inhibit the production of phenolics. Our results demonstrated that supplementing the culture medium with PSK stimulated protoplast division and aggregates formation of hypocotyl- and MC-protoplast-derived cells. It should be noted that in PSK-free culture variant media, cell divisions were not observed. Also, applying PSK to callus multiplication medium enhanced the formation of embryogenic tissue. Similar stimulation of protoplast culture development as a result of PSK application was observed in *Beta vulgaris* [55], *Oryza sativa* [22], *Brassica oleracea* [19, 20], and *Daucus* ssp. [54]. Protoplast isolation is a stress-inducing procedure that can generate active oxygen species [44, 62]. Therefore, applying exogenous polyamines such as PUT seems to overcome this problem. Additionally, polyamines impact maintaining protoplast viability, increase mitotic activity and shoot regeneration [29]. Nevertheless, the application of PUT had no significant effect on the plating efficiency (number of cell aggregates formed) in MC- and hypocotyl-derived protoplast cultures of Tartary buckwheat. Comparable to our results, also in protoplast cultures of *Nigella damascena*, the application of PUT did not significantly affect plating efficiency [63]. We also implemented urea-type synthetic cytokinin (CPPU) that, according to the literature, participates in cell division and expansion [64]; induction of embryogenic callus [65] and shoot formation [66]. The supplementation of PSK-rich BM medium with PUT or CPPU or a combination of both enhanced the development of protoplast cultures and somatic embryos formation but did not increase the plating efficiency. This indicates that protoplast cultures of Tartary buckwheat are able to develop (i.e. to undergo the way from first mitotic to microcallus formation) only in the presence of PSK.

A common problem in protoplast and tissue cultures is oxidative browning of the culture media and tissue [30]. As mentioned in the background, phenolic compounds can block developmental processes in in vitro cultures. For our study, applying AIP (reversible inhibitor of PAL) in the MC- and hypocotyl-derived protoplast cultures did not prevent tissue browning or influence plating efficiency. In contrast to our results, *Ulmus americana*-derived protoplasts isolated from callus cultured in the presence of AIP were characterised by a higher rate of cell divisions and developed cell walls faster [36]. However, later studies showed, that AIP had no impact on the growth and development of protoplast-derived callus and shoots [60]. Another common compound applied to decrease tissue browning is PVP, which absorbs, among other compounds, phenolics [67]. Our study recorded visible reduction of tissue browning in protoplast-derived microcallus originating from hypocotyls. Nevertheless, the reduction of tissue browning was not associated with an increase in plating efficiency. Similarly to our observation in *Cyamopsis tetragonoloba* [31] and *Vitis* [32], the application of PVP did not prevent the browning of the culture media but reduced it to a low level.

So far, immature embryos, hypocotyls, and cotyledons of Tartary buckwheat were successfully applied to plant regeneration [9, 14]. According to Wang et al. [68], hypocotyl explants were better source material than cotyledons for Tartary buckwheat regeneration. Similarly, the regeneration of plants via somatic embryogenesis from hypocotyl explants was achieved by Han et al. [2]. In contrast to the presented examples, we did not observe plant regeneration in protoplast cultures originating from hypocotyls. Similarly to our results, Lachmann and Adachi [43] only reported about callus formation in hypocotyl-derived protoplast cultures. According to Pasternak et al. [69], the disadvantage of hypocotyls application as a source for protoplast isolation and cultures is rapidly increasing in cell ploidy level. For example, in *Cucumis sativus*, polysomaty was present in the hypocotyls and roots at the early stages of tissue differentiation. Moreover, the polysomatic nature of Tartary buckwheat plants [70] may explain the supposed polyploidisation of the tissue originating from hypocotyl protoplast cultures and lack of regeneration ability. Additionally, our histological observations revealed the presence of irregularity in shape nuclei and more than one nucleoli. In non-morphogenic calli of *Beta vulgaris*, nuclei with irregular shapes and many nucleoli were observed, indicating polyploidy and aneuploidy [71]. A correlation between cell polyploidisation and instability of nuclei size and DNA content was found in the callus of *Allium fistulosum* [72]. Morphological characteristics of microcalli originating from hypocotyl protoplast cultures apparently explain this tissue’s loss of regeneration capacity.

Due to the totipotency of plant cells, i.e. the possibility of their reprogramming from a differentiated state of a cell to a dedifferentiated state, plants are characterised by a high ability to regenerate, including when they are cultured in vitro [73]. Cellular reprogramming is associated with changes in transcriptome, which plays a significant role in the regulation of plant differentiation and plant development [74]. According to these views, we speculated that applying protoplast culture technology may result in the dedifferentiation of the NC cells of Tartary buckwheat, loss of their characteristic features, and reprogramming into embryogenically determined cells. The results demonstrate that the level of dedifferentiation of donor tissue during the removal of the cell wall and cell division is significant in protoplast regeneration. Yang et al. [75] hypothesised that non-embryogenic callus cells might have the ability to differentiate into embryogenic cells. Contrarily, Fehér [76] mentioned that protoplasts often retain the characteristic features of progenitor cells, which should be lost in the presence of hormones. Studies by Faraco et al. [77] showed that protoplasts retain their tissue- and cell-specific features during transient expression assay. These authors showed gene expression in protoplasts originating from the epiderma of petal and in the intact flower. Additionally, Sheen [78] pointed out that despite cell wall removal, protoplasts retain physiological responses and cellular activities as intact plants. Therefore, we may suppose that applied conditions and PGRs in protoplast cultures media were insufficient to complete cell dedifferentiation to embryogenically determined cells. As it was demonstrated by Betekhtin et al. [70], NC is composed mainly of parenchymatous cells, with inhibited capacity for morphogenesis. In our study, calli originating from NC-derived protoplast cultures consist of the same types and structures of the cells, characterised by friable structure, rapid growth, and lack of ability for regeneration. The irregular shaped nuclei of the protoplast-derived calli may indicate an increased amount of nuclear DNA. Similar observations were demonstrated for *Daucus carota* [79] and *Rosa hybrida* [89]. The authors noted a lack of regeneration after using as protoplast source non-embryogenic callus or non-embryogenic cell suspension cultures.

The cells of calli originating from MC-derived protoplast cultures were characterised by the abundance of embryogenic cells as described by Verdeil et al. [80]. The same features point out the ability to regenerate and strongly confirm the morphogenic character of the protoplast-derived tissue. According to Betekhtin et al. [70] MC is an excellent example of maintaining the regeneration potential due to genetic and cytogenetic stability in long-term cultivation. Transferring the calli originating from MC-derived protoplast cultures to a regeneration medium with BAP and KIN (supplemented with PVP) stimulates somatic embryogenesis and organogenesis with the following conversion into plants. In similar conditions, plant regeneration via somatic embryogenesis was demonstrated by Wang et al. [68] from hypocotyl explants. In summary, we suppose that the success of regeneration might depend on the genotype used in the study. The genotype-dependence in the development of protoplast cultures and their ability to regenerate was noted for *Brassica oleracea* [19, 25, 29, 48], *Daucus carota* [21, 54], *Beta vulgaris* [55] and *Musa ssp.* [81].

## Conclusions

The present study demonstrated a successful approach for callus regeneration from hypocotyl- and, for the first time, plant regeneration from morphogenic callus-derived protoplasts of Tartary buckwheat. We demonstrated high cell colony and microcalli formation efficiency could be induced after protoplast embedding in agarose matrix and supplementing a culture medium with PSK. The presented protoplast-to-plant system enables using protoplasts as a model material for genetic engineering, i.e. genetic transformation of buckwheat to improve this agronomically important crop. This protocol can be helpful for precise genome editing using Cas9 ribonucleoprotein complexes. In addition, practical applications implemented for protoplast isolation, culture, and regeneration can be used in somatic hybridization between different *Fagopyrum* species.

### Methods

#### Plant materials

As a protoplasts source, one line of the NC (Fig. 2a), four lines of the MC (MC1, MC2, MC4, NL2018; Fig. 2b-e) and etiolated hypocotyls of in vitro grown seedlings were used (Fig. 2f). The callus lines were obtained from the immature embryo of *F. tataricum* and maintained in the dark at 26 ± 1^o^C on RX medium as described by Betekhtin et al. [70]. RX medium contained the mineral salts according to Gamborg’s medium [82] (B5; Duchefa, The Netherlands), 2 g L^− 1^ N-Z-amine A (Sigma-Aldrich, USA), 2.0 mg L^− 1^ 2,4-dichlorophenoxyacetic acid (min. 98%) (2,4-D; Sigma-Aldrich), 0.5 mg L^− 1^ indole-3-acetic acid (IAA; Sigma-Aldrich), 0.5 mg L^− 1^ α-naphthalene acetic acid (NAA; Sigma-Aldrich), 0.2 mg L^− 1^ kinetin (KIN; Sigma-Aldrich), 25 g L^− 1^ sucrose (POCH, Poland) and 7 g L^− 1^ phyto agar (Duchefa) [70]. The NC and MC callus lines were subcultured every two weeks. Aseptic hypocotyls were produced in vitro from seeds (obtained from the collection of the N. I. Vavilov Institute of Plant Genetic Resources, Saint Petersburg, Russia) surface sterilised using a two-step procedure. First, seeds were dipped in 70% (*v/v*) ethanol for 30 s, then transferred to 0.1% (*v/v*) solution of fungicide Gwarant (Arysta, France) with one drop of Tween 20 (Duchefa) and placed on a gyratory shaker (160 rpm) and finally immersed in a 20% (*w/v*) solution of chloramin T (sodium N-chlorotoluene-4-sulphonamide; Chempur, Poland) with 800 mg L^− 1^ cefotaxime disodium (Duchefa) and one drop of Tween 20 (30 min each step). After each step, the seeds were dipped in 70% ethanol for 30 s. Then the seeds were washed three times in sterile distilled water for 5 min each and left in the sterile distilled water overnight. On the next day, the washes in sterile water were repeated, the seeds were air-dried on a sterile filter paper and about eight seeds per Petri dish (Ø9 cm) were placed on solid Murashige and Skoog [83] medium with vitamins (MS; Duchefa) supplemented with 30 g L^− 1^ sucrose and 7 g L^− 1^ plant agar (Duchefa) and maintained at 26 ± 1^o^C in the dark for 10 days for seeds germination.

### Protoplast isolation and culture

Protoplasts were isolated from 1-2-week-old callus and hypocotyls excised from 10-day-old seedlings, using the protocol of Grzebelus et al. [21] with some modifications. For protoplast isolation from callus 1 g of plant material was placed in a glass Petri dish (Ø9 cm) with preplasmolysis solution consisting of 0.6 M mannitol (Sigma-Aldrich) and 5 mM CaCl_2_ (Sigma-Aldrich), cut into small pieces and then incubated for 1 h in the dark at 26 ± 1^o^C. Release of protoplasts took place overnight (16 h) at 26 ± 1^o^C, with gently shaking (30–40 rpm) in the enzyme mixture consisting of 1% (*w/v*) cellulase Onozuka R-10 (Duchefa), 0.1% pectolyase Y-23 (Duchefa), 20 mM 2-(N-Morpholino) ethanesulfonic acid (MES, Sigma-Aldrich), 5 mM MgCl_2_ × 6H_2_O (POCH), and 0.6 M mannitol, pH 5.6, filter-sterilised (0.22 µm; Millipore, Billerica, MA, USA). In the case of hypocotyls 1 g of tissue was cut into 1 cm pieces in length and then cut longitudinally in preplasmolysis solution (0.5 M mannitol). The tissue was macerated in the enzyme mixture containing of 1% cellulase Onozuka R10, 0.6% macerozyme R10 (Duchefa), 0.1–0.25% driselase® (Sigma-Aldrich), 20 mM MES, 5 mM MgCl_2_ × 6H_2_O and 0.6 M mannitol, pH 5.6, filter-sterilised (0.22 µm). The released protoplasts were separated from undigested tissue by filtration through a 100 µm nylon sieve (Millipore) and then centrifuged at 100 g for 5 min. Pellets were re-suspended in 0.5 M or 0.6 M sucrose with 1 mM MES for callus and hypocotyls, respectively, overlaid with W5 solution [84] and centrifuged at 145 g for 10 min. Protoplasts localised in the interphase between sucrose/MES and W5 solution were collected into a new tube and washed twice by centrifugation at 100 g for 5 min in W5 solution and then once in the culture medium. All protoplast culture media were based on the CPP medium according to Dirks et al. [85] and consisted of macro-, micro-elements and organic acids according to Kao and Mychayluk [86] (KM; Duchefa), vitamins according to B5 medium [82] (Duchefa), 74 g L^− 1^ glucose (POCH) and 250 mg L^− 1^ casein enzymatic hydrolysate (Sigma-Aldrich), pH = 5.6, filter sterilised. After purification the protoplasts were suspended in 1 ml of the culture medium and their yield was determined using a Fuchs-Rosenthal haemocytometer (Heinz Herenz, Germany). The working density before cell embedding was adjusted to 8 × 10^5^ or 5 × 10^5^ cells per ml for callus- and hypocotyl-derived protoplasts, respectively. For protoplast embedding the filter-sterilised solution of 1.2% (*w/v*) SeaPlaque agarose (Duchefa) or filter-sterilised solution of 2.8% (*w/v*) alginic acid sodium salt (Sigma-Aldrich) were applied according to the protocol of Grzebelus et al. [55] and Grzebelus et al. [54], respectively. In the case of agarose embedding three to four 50 µl-aliquots of the protoplast/agarose mixture were dropped into a Petri dish (Ø 6 cm) and after solidification of the agarose beads (app. 15 min) 4 ml of the culture medium was added. For NC-derived protoplast cultures, the culture medium was supplemented with six different combinations of auxins and cytokinins, as shown in Fig. 4a. For MC- and hypocotyl-derived protoplast cultures the culture medium was supplemented with BAP 1.0 mg L^− 1^ and NAA 2.0 mg L^− 1^ and hereinafter referred to as basal medium (BM) for protoplast cultures. BM was additionally supplemented in different combinations with 100 nM phytosulfokine-α (PSK; PeptaNova GmbH, Germany), 8.0 mg L^− 1^ Putrescine (PUT; Sigma-Aldrich), 0.5 mg L^− 1^ N-(2-chloro-4-pyridyl)-N’-phenylurea (CPPU; Sigma-Aldrich), 0.025% or 0.05% polyvinylpyrrolidone (PVP, MW 40,000; Sigma-Aldrich) and 10 µM 2-aminoondane-2-phosphonic acid (AIP; Chemat, Poland) as shown in Figs. 5 and 6. To prevent endogenous bacterial contaminations, all protoplast culture media contained 300 mg L^− 1^ ticarcillin disodium (Duchefa) or 200 mg L^− 1^ cefotaxime disodium (Duchefa) in callus- or hypocotyl-derived protoplast cultures, respectively. Protoplast cultures were incubated at 26 ± 1^o^C in the dark. After 10 days of culture, the medium with all supplements was replaced by a fresh one.

### Histological analysis of protoplast-derived callus

Histological analyses were performed according to Betekhtin et al. [70] with minor modifications. Samples of microcalli obtained from two-month-old protoplast cultures were fixed in 4% paraformaldehyde (PFA, POCH) and 1% glutaraldehyde (GA, POCH) in 0.1 M phosphate buffered saline (PBS, pH 7.2) overnight at 4^o^C. Subsequently, the samples were rinsed in PBS, dehydrated in increasing ethanol concentrations, and then embedded in LR White resin (Polysciences, PA). Samples were cut into 1.5 μm thick sections using a Leica EM UC6 ultramicrotome (Leica Biosystems, Germany), placed on glass slides coated with poly-L-lysine (Gerhard Menzel, Germany), stained with 0.05% Toluidine Blue O (Sigma-Aldrich) and mounted under a coverslip in Euparal medium (Sigma-Aldrich). The stained sections were examined under an Olympus BX43F microscope (Olympus LS, Tokyo, Japan) equipped with the Olympus XC50 digital camera.

### Plant regeneration from protoplast-derived tissue

After about two months of protoplast culture, protoplast-derived callus in agarose beads were transferred to a callus multiplication medium (CM) consisting of macro-, micro-elements and vitamins according to MS medium [83], 2 g L^− 1^ N-Z-amine A, 2.0 mg L^− 1^ 2,4-D, 0.2 mg L^− 1^ KIN, 100 nM PSK, 30 g L^− 1^ sucrose and 3 g L^− 1^ phytagel (Sigma-Aldrich). The cultures were maintained at 26 ± 1^o^C in the dark and subcultured every three to four weeks. For plant regeneration, callus clumps or PECCs were transferred onto the regeneration medium (RM) containing macro- and micro-elements as in MS medium [83], 2.0 mg L^− 1^ BAP, 1.0 mg L^− 1^ KIN, 0.0025% PVP, 30 g L^− 1^ sucrose, 3 g L^− 1^ phytagel and cultured in a growth room at 28 ± 2^o^C with a 16/8 h (light/dark) photoperiod, under light intensity of 55 µmol m^− 2^ s^− 1^, and subcultured every three weeks. During three subcultures callus clumps and PECCs converted into bipolar and cotyledonary embryos, respectively. Small rooting shoots were transferred to a medium without PGRs containing macro-, micro-elements and vitamins according to MS [83], 30 g L^− 1^ sucrose and 3 g L^− 1^ phytagel and maintained in a growth room at 25 ± 2^o^C with a 16/8 h (light/dark) photoperiod, under a light intensity of 55 µmol m^− 2^ s^− 1^.

### Numerical data collection and statistical analysis

The yield of protoplast isolation, protoplast viability and plating efficiency were determined. The protoplast yield was expressed as the number of protoplasts per gram of fresh weight of source material. Protoplast viability was assessed by staining the cells just after embedding in agarose beads with fluorescein diacetate (FDA; Sigma-Aldrich) according to Grzebelus et al. [21]. the viability of protoplasts was determined as a number of protoplasts with apple-green fluorescence per total number of observed cells (×100). Pre-mitotic symptoms in 10-day-old cultures of NC-derived protoplasts were expressed as the number of cells enlargement in size and with reorganized cytoplasm per total number of observed cells (×100). Plating efficiency was evaluated in 10-day-old cultures and expressed as the number of cell aggregates per total number of observed undivided cells and cell colonies (×100). Observations were performed using an Axiovert S100 inverted microscope (Carl Zeiss, Germany) equipped with a filter set appropriate for FDA detecting (λ_Ex_ = 485 nm, λ_Em_ = 515 nm).

At least two to five independent protoplast isolation experiments with a single treatment represented by three-four Petri dishes were carried out as biological repetitions. Microscopic observations were carried out on 100–200 cells per Petri dish. Means and the standard error of the means were calculated. Data were subjected to one-way analysis of variance (ANOVA) using Statistica 13 (TIBCO Software Inc., USA). Tukey’s posthoc test was used to determine significant differences between the means.

## Data Availability

All data generated or analysed during this study are included in this published article.

## Abbreviations

2,4-D: 2,4-dichlorophenoxyacetic acid
AIP: 2-aminoondane-2-phosphonic acid
B5: Gamborg medium
BAP: 6-benzylaminopurine
BM: basal medium
CM: callus multiplication medium
CPPU: N-(2-chloro-4-pyridyl)-N’-phenylurea
FDA: Fluorescein diacetate
GA: glutaraldehyde
KIN: kinetin
KM: Kao and Michayluk medium
MC: morphogenic callus
MS: Murashige and Skoog medium
NAA: α-naphthalene acetic acid
NC: non-morphogenic callus
PAL: phenylalanine ammonia-lyase
PBS: phosphate buffered saline
PCC: phenolic-containing cells
PECCs: proembryogenic cell complexes
PEMs: pro-embryogenic masses
PFA: paraformaldehyde
PGRs: plant growth regulators
PSK: phytosulfokine-α
PUT: putrescine
PVP: polyvinylpyrrolidone
RM: regeneration medium
